# The relationship between skeletal muscle mass to visceral fat area ratio and metabolic dysfunction-associated fatty liver disease subtypes in middle-aged and elderly population: a single-center retrospective study

**DOI:** 10.3389/fnut.2023.1246157

**Published:** 2023-11-08

**Authors:** Mengchen Xing, Yanlan Ni, Ye Zhang, Xiaoqian Zhao, Xin Yu

**Affiliations:** ^1^Department of Thyroid, Breast, and Gastrointestinal Surgery, The Affiliated Wuxi People’s Hospital of Nanjing Medical University, Wuxi People’s Hospital, Wuxi Medical Center, Nanjing Medical University, Wuxi, China; ^2^Department of Minimally Invasive Laparoscopy, The Affiliated Wuxi People’s Hospital of Nanjing Medical University, Wuxi People’s Hospital, Wuxi Medical Center, Nanjing Medical University, Wuxi, China; ^3^Emergency Intensive Care Unit, The Affiliated Wuxi People’s Hospital of Nanjing Medical University, Wuxi People’s Hospital, Wuxi Medical Center, Nanjing Medical University, Wuxi, China; ^4^Department of Hepatobiliary Surgery, The Affiliated Wuxi People’s Hospital of Nanjing Medical University, Wuxi People’s Hospital, Wuxi Medical Center, Nanjing Medical University, Wuxi, China

**Keywords:** skeletal muscle mass to visceral fat area ratio, metabolic dysfunction-associated fatty liver disease subtypes, gender, fibrosis, middle-aged, elderly

## Abstract

**Background:**

It has been reported that decreased muscle mass combined with excessive visceral adipose tissue are significantly correlated with the risk of non-alcoholic fatty liver disease (NAFLD). However, it has not been explored among populations with metabolic dysfunction-associated fatty liver disease (MAFLD) subtypes. We aimed to investigate whether appendicular skeletal muscle mass to visceral fat area ratio (SVR), an indicator of sarcopenic obesity, influences on the risk of MAFLD subtypes and its hepatic condition in middle-aged and elderly population.

**Methods:**

A total of 4,003 middle-aged and elderly subjects were finally enrolled in this single-center retrospective study. Abdominal ultrasonography was employed for hepatic steatosis diagnosis. Participants were divided into four groups: diabetes-MAFLD, overweight/obese-MAFLD, lean-MAFLD and no MAFLD. Appendicular skeletal muscle mass as well as visceral fat area (VAF) was estimated by bioimpedance analysis measurements. Liver fibrosis was defined as a Fibrosis-4 index (FIB-4) and the NAFLD Fibrosis Score (NFS). Multivariate logistic regression analysis was performed to estimate the odds ratio and 95% confidence interval between SVR and MAFLD subtypes/hepatic condition stratified by sex.

**Results:**

Participants with MAFLD subtypes had a significant lower value of SVR compared with those without MAFLD (*P*<0.001), while high quartiles of FIB-4 and NFS also showed a decreasing value of SVR in comparison with its lower quartiles (*P*_for trend_<0.001). The lowest quartile of SVR increased the prevalence of MAFLD subtypes [adjusted OR (95%CI): 2.96 (1.48 ~ 5.93) _male_ /3.30(1.46 ~ 7.46) _female_ for diabetes-MAFLD, 1.91(1.26 ~ 2.88) _male_ /4.48(1.91 ~ 10.49) _female_ for overweight/obese-MAFLD and 4.01(1.46 ~ 10.98) _male_/2.53(1.19 ~ 5.37) _female_ for lean-MAFLD groups] compared with the highest quartile of SVR (all *P*_for trend_<0.001). Besides, the interaction effect of gender on the relationship between SVR and MAFLD subtypes was statistically significant (all *P*_for interaction_<0.001).Restricted cubic spline indicated an inverse association between SVR and the risk of MAFLD subtypes with linearity (all *P* for non-linearity>0.05). The lowest quartile of SVR also increases the risk of MAFLD fibrosis in both males and females.

**Conclusion:**

Our study concluded that a decrease in SVR (appendicular skeletal muscle mass divided by visceral fat area) is significantly associated with an increased prevalence of developing MAFLD subtypes and liver fibrosis in middle-aged and older persons of both genders.

## Introduction

Metabolic associated fatty liver disease (MAFLD), previously known as NAFLD, is a liver disease related to metabolic dysfunction and is the most common chronic liver disease worldwide. It is estimated that 24% of adults are affected by NAFLD, posing a serious threat to human health (1). In contrast to NAFLD, MAFLD does not necessitate the exclusion of other sources of liver disease, like overindulging in alcohol or viral hepatitis (2, 3). An international expert consensus recently concluded that “MAFLD” was a more suitable term to describe liver disease caused by metabolic disorders, and subsequently released a set of diagnostic criteria to facilitate the accurate, comprehensive and straightforward diagnosis of MAFLD (3). MAFLD is a multi-system disorder that increases the risk of liver-specific complications, as well as other health concerns such as cardio-metabolic morbidity and mortality (4–7). To gain a better comprehension of how to identify those at high risk and create successful treatments for the illness, Further investigation is necessary to comprehend the diverse elements that contribute to the etiology and pathogenesis of this complex liver condition.

Excess weight, particularly abdominal obesity, is a major risk factor for MAFLD (8, 9). VFA (Visceral Fat Area) is a reliable and reproducible measure of abdominal obesity, and is linked to a greater risk of metabolic syndrome (MetS) and MAFLD than BMI (Body Mass Index) and WC (Waist Circumference) as indicators of adiposity (10). Furthermore, decreased muscle mass, known as sarcopenia, has also been identified as a risk factor for MAFLD, as skeletal muscle influences glucose disposal and insulin resistance (11–13). Sarcopenic obesity, a combination of low muscle mass and high visceral fat, has a substantial influence on metabolism and increases the risk of MAFLD. It has been suggested that alterations in body composition, such as an increase in visceral fat and a decrease in skeletal muscles due to muscular protein degradation in the context of obesity-related chronic inflammation, may be linked to the onset and progression of NAFLD. Furthermore, abnormal body composition has been found to be a significant factor influencing the pathophysiology and prognosis of hepatic conditions (14). By using bioelectrical impedance, it is possible to measure the appendicular skeletal muscle mass and intra-abdominal visceral fat area, and the SVR index, which is an indicator of sarcopenic obesity, can be derived from these measurements.

It has been established that SVR has a strong correlation with the probability of having NAFLD and the worsening of hepatic conditions. To date, few studies have investigated the impact of SVR on the risk of developing different MAFLD subtypes and its hepatic condition among the health check-up population. Therefore, this study aimed to explore the gender-specific associations between SVR and the risk of MAFLD subtypes and its hepatic condition in middle-aged and elderly populations.

## Materials and methods

### Study population

**Figure 1:**
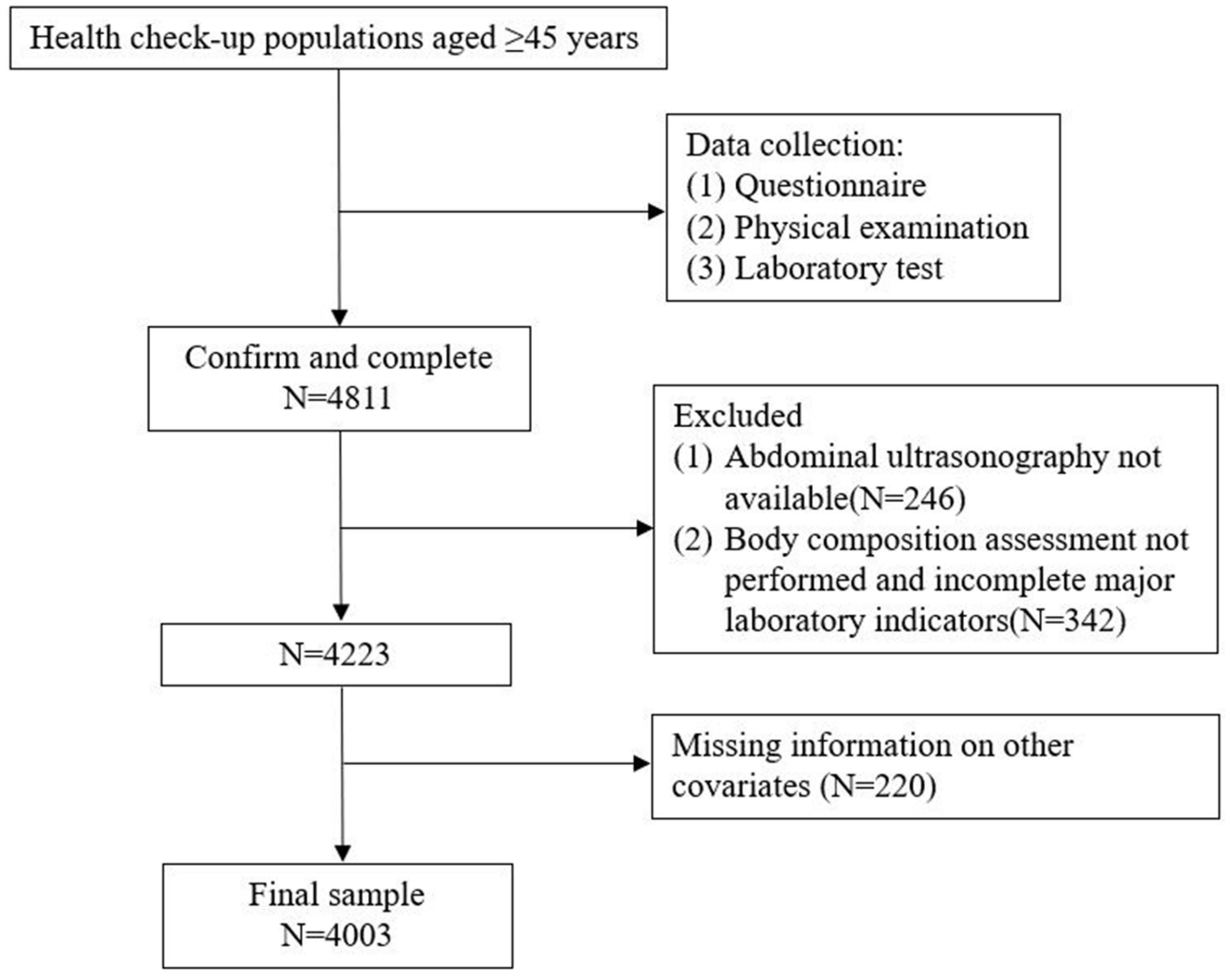
Study flow chart illustrated the flow of the participants included in this study.

Individuals aged 45 years or above who attended annual health examinations in the health check-up center of Wuxi People’s Hospital Affiliated to Nanjing Medical University were included. This retrospective study originally enrolled 4,811 middle-aged and elderly population. Participants were excluded if they had incomplete medical information mainly including bioelectrical impedance analyzer (BIA) measurements and abdominal ultrasound. Excluding the participants mentioned (Figure 1), 4,003 individuals were finally included in the study, comprising of 2,420 males and 1,583 females aged between 45 and 91 years. Demographic data comprised of age, gender, and cigarette/alcohol use; smoking was classified as smoking three or more cigarettes daily in a year, and alcohol consumption of at least three times a week for at least 12 months.

This retrospective study was performed in compliance with the Declaration of Helsinki and was permitted by the Health Examination Center of Wuxi People’s Hospital Ethics and Research Committee (approval number KY23150). Personal data was anonymized in order to protect the privacy of patients; statistical analysis was conducted in a confidential manner and was only utilized for scientific objectives. Therefore, the necessity for informed consent was waived.

### Body composition and anthropometric measurements

Assessment of the body composition of all participants was done using the segmental multifrequency bioelectrical impedance analysis system (InBody 4.0, InBody Co., South Korea). The validated equation of Janssen et al. was used to calculate the skeletal muscle mass of each subject, which is calculated as (15): skeletal muscle mass (kg) = [(height^2^/BIA resistance * 0.401) + (gender*3.825) + (age * − 0.071)] + 5.102, where height is in centimeters, BIA resistance is in ohms, gender is coded as 1 for male and 0 for female, and age is in years. Appendicular skeletal mass (ASM) is the sum of lean muscle mass in the upper and lower limbs. The skeletal muscle mass to visceral fat area ratio (SVR) is calculated by dividing ASM by the visceral fat area. Body weight, height, and waist circumference (WC) were measured by trained nurses, and Body Mass Index (BMI) was calculated using the formula: BMI = body weight (kg)/height (m)2. WC was measured to the nearest 0.1 cm around the horizontal level at the high point of the iliac crest.

### Laboratory and clinical measurements

Blood specimens (10−15 mL) were collected from the antecubital vein of participants after at 12-h overnight fast. The serum was placed at room temperature for 30 min and centrifuged at 3000 rpm for 10 min. Laboratory measurements included fasting blood glucose (FBG), triglycerides (TGs), total cholesterol (TC), low-density-lipoprotein cholesterol (LDL-C), high-density-lipoprotein cholesterol (HDL-C), serum aspartate aminotransferase (AST), alanine aminotransferase (ALT), white blood cells (WBC), neutrophils (NE), and lymphocytes (LY). All blood samples were tested within 24 h in medical laboratory center of Wuxi People’s Hospital Affiliated to Nanjing Medical University.

Diabetes mellitus was defined as fasting blood glucose ≥ 100 mg/dL or currently receiving antidiabetic medication therapy (16), while hypertension was defined as systolic blood pressure ≥ 140 mmHg or diastolic blood pressure ≥ 90 mmHg, or receiving anti-hypertension treatment at present (17). dyslipidemia defined as two or more of these four criteria: fasting serum triglyceride ≥ 1.70 mmol/L, TC ≥ 5.20 mmol/L, LDL cholesterol ≥ 3.12 mmol/L, or HDL cholesterol ≤ 0.91 mmol/L.

### Assessment of MAFLD and liver fibrosis

MAFLD was identified when hepatic steatosis was present, and at least one of the following criteria (3): being overweight or obese (BMI ≥ 23 kg/m^2^), having type 2 diabetes mellitus, or exhibiting metabolic dysregulation. Metabolic dysregulation was defined by the occurrence of at least two of the following risks for metabolic disorders (18): ① waist circumference ≥ 90/80 cm in Asian men and women; ② blood pressure ≥ 130/85 mmHg or specific drug treatment for controlling blood pressure; ③ plasma triglyceride ≥ 1.7 mmol/L or specific drug treatment; ④ plasma high-density lipoprotein cholesterol < 1.0 mmol/L for men and < 1.3 mmol/L for women or specific drug treatment; ⑤ prediabetes with fasting glucose levels between 5.6 to 6.9 mmol/L; ⑥ homeostasis model assessment of insulin resistance (HOMA-IR) score ≥ 2.5.

Hepatic steatosis was measured using abdominal high-resolution ultrasonography (SIMENS ACUSON S2000 ABVS) after 12 h fasting. Well-trained radiologists conducted the ultrasonography and diagnosed the hepatic steatosis according to the characteristics of ultrasonic diagnosis satisfied the following abnormal abdominal ultrasound images (19–21): The near field echo of liver increased diffusely (“bright liver “), and the echo of liver was larger than that of kidney or spleen; vascular blurring; Poor visibility of the posterior right lobe due to deep attenuation.

In this research, participants with MAFLD were categorized into three groups (22, 23). To begin with, we identified the diabetes-MAFLD group depending on the presence of DM regardless of BMI. Subsequently, in individuals without DM, we classified MAFLD subgroups based on BMI: ①overweight/obese (BMI ≥ 23 kg/m^2^) or ②lean or normal weight (BMI < 23 kg/m^2^). In the end, the study population was divided into four subgroups: no MAFLD, diabetes-MAFLD, overweight/obese-MAFLD, and lean-MAFLD.

The fibrotic burden of the liver was evaluated using the fibrosis-4 index (FIB-4) or NAFLD fibrosis score (NFS). Previous research has demonstrated that the FIB-4 index has a similar overall diagnostic performance for diagnosing MAFLD fibrosis, and it has also been shown to have a better diagnostic performance for fibrosis in all MAFLD subtypes (24). In this study, both FIB-4 index and NFS were used to assess the liver fibrosis by the following equation (25): FIB-4 index = age (years)*AST(U/L)/ [PLT (10^9^/L) * ALT(U/L)^1/2^], NFS = − 1.675 + 0.037 * age + 0.094 * BMI + 1.13 * Prediabetes/diabetes + 0.99 * (AST / ALT) - 0.013 * PLT - 0.66 * Alb. The cut-off values of FIB-4 index and NFS in diagnosing fibrosis were at least 1.3 and − 1.455, respectively (26).

### Statistical analysis

All statistical analyzes were performed using SPSS 26.0 software and STATA 17.0 software. Continuous variables are reported as mean ± SD or median with interquartile ranges according to the evaluation of the normal distribution by Shapiro–Wilk test, and categorical variables are presented as the numbers(n) with percentages (%). Comparisons of baseline characteristics and laboratory parameters specified by gender were conducted using the Student’s *t*-test or Mann–Whitney *U*-test for continuous variables, and the chi-square test for categorical variables as appropriate. Restricted cubic spline model was performed to estimate the association between SVR and MAFLD subtypes among gender in a fully adjusted model. The knots were located at the 5th, 35th, 65th, and 95th percentiles. Additionally, a multivariable regression model was used to evaluate the relationship between SVR quartiles (based on gender) and MAFLD subtypes and its fibrosis by sex, with the fourth quartile as the reference group, adjusting for potential confounders. Model 1 adjusted age, smoking, drinking, WC, and BMI. Model 2 adjusted Model 1 plus FBG, hypertension and dyslipidemia. Model 3 adjusted model 2 plus ALT, AST, WBC, NE and LY. Adjusted odds ratios (ORs) were presented with 95% confidence interval (CI). *P* for trend was evaluated for linear trend test using the median value of SVR as a continuous variable in the adjusted models. The interactions effect of gender on the relationship between SVR and MAFLD subtypes were assessed by including stratification analysis and interaction tests in the regression model (*P*_for interaction_). A two-tailed *p*-value < 0.05 was considered statistically significant.

## Results

### Baseline characteristics of participants

**Figure 2:**
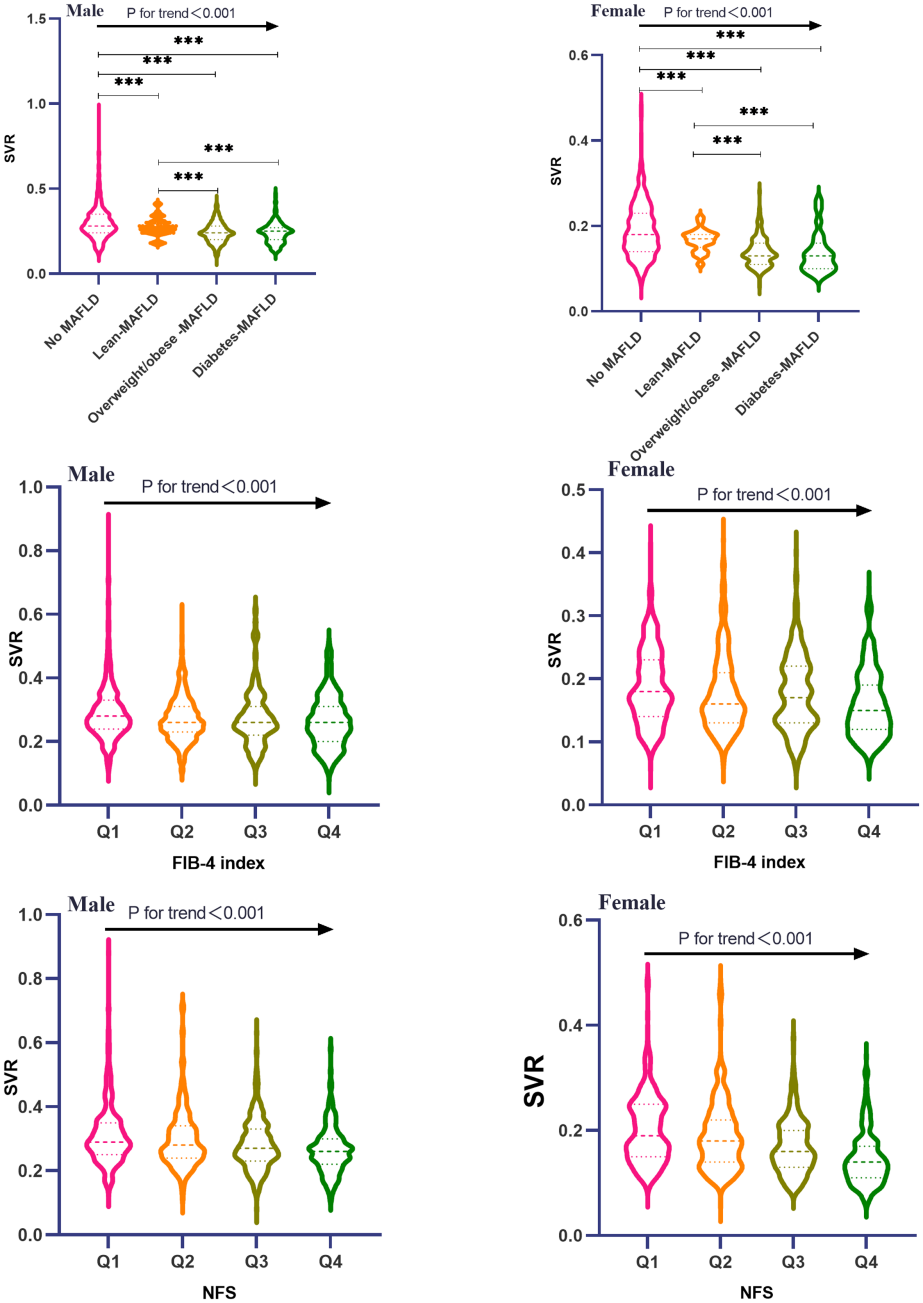
Comparison of SVR among all MAFLD subtypes and FIB-4/NFS quartiles stratified by sex.

Baseline characteristics of 4,003 participants were presented in Table 1, Prevalence of lean-MAFLD, overweight/obese-MAFLD and diabetes-MAFLD stratified by gender was 4.4% (106), 30.2% (730) and 7.8% (188) for males, and 5.6% (88), 19.0% (301) and 7.3% (115) for females. Significant differences were existed in different variables including age, smoking, drinking, waist circumference, BMI, VFA, ASM, SBP, DBP, hypertension, FPG, diabetes, TG, TC, HDL-C, LDL-C, AST, ALT, FIB-4 index, NFS, WBC, NE, LY among groups (all *p*-value <0.05). Moreover, baseline characteristics of the participants were also compared according to SVR quartiles (Table 2), both males and females in the lowest quartile (Q1) of SVR tended to be older, and had the highest level of WC, BMI, ALT, AST, FIB-4, NFS, WBC, NE and LY than those in another three quartiles (*P*_for trend_<0.05). Furthermore, there were a higher proportion of subjects of hypertension, diabetes and dyslipidemia in the first quartile of SVR compared to the higher SVR quartile(*P*_for trend_<0.05). Besides, different MAFLD subtypes had a higher SVR value than those without MAFLD (*P*<0.001; Figure 2). Participants with lean-MAFLD had an increasing value than those with overweight/obese MAFLD or diabetes-MAFLD (*P*<0.001). there was no significant difference between overweight/obese MAFLD group and diabetes-MAFLD group in SVR values (*P*>0.05). Additionally, both males and females had a decreasing value of SVR in the higher quartile of FIB-4 index and NFS (*P*_for trend_<0.001).

**Table 1:** Baseline characteristics of the study population with different MAFLD subtypes according to sex (*n* = 4,003).

Characteristics	Male (*n* = 2,420)				*P*-value	Female (*n* = 1,583)				*P*-value
	No MAFLD (*n* = 1,396)	Lean-MAFLD (*n* = 106)	Overweight/obese–MAFLD (*n* = 730)	Diabetes-MAFLD (*n* = 188)		No MAFLD (*n* = 1,079)	Lean-MAFLD (*n* = 88)	Overweight/obese -MAFLD (*n* = 301)	Diabetes-MAFLD (*n* = 115)	
Age (years)	58.68 ± 9.77	58.04 ± 6.80	55.94 ± 7.39	58.11 ± 7.42	<0.001	55.06 ± 8.22	57.35 ± 7.65	56.69 ± 7.37	63.39 ± 9.68	<0.001
Smoking (*n*, %)	738 (52.9)	67 (63.2)	450 (61.6)	126 (67.0)	<0.001	9 (0.8)	6 (6.8)	7 (2.3)	11 (9.6)	<0.001
Drinking (*n*, %)	464 (33.2)	62 (58.5)	252 (34.5)	69 (36.7)	<0.001	23 (2.1)	6 (6.8)	21 (7.0)	8 (7.0)	<0.001
WC (cm)	87.08 ± 7.12	84.33 ± 4.72	95.78 ± 7.51	93.95 ± 7.87	<0.001	80.97 ± 6.81	81.63 ± 3.21	89.84 ± 6.99	88.15 ± 7.76	<0.001
BMI (kg/m^2^)	24.20 ± 2.43	21.99 ± 0.79	27.16 ± 2.45	26.67 ± 2.81	<0.001	22.81 ± 2.48	22.11 ± 0.76	26.19 ± 2.45	25.82 ± 2.86	<0.001
VFA (cm^2^)	80.09 ± 24.69	72.92 ± 15.54	104.38 ± 27.99	102.21 ± 27.22	<0.001	90.45 ± 30.42	89.17 ± 15.31	126.78 ± 29.71	124.74 ± 34.96	<0.001
ASM (kg)	22.13 ± 2.78	19.83 ± 2.05	23.78 ± 2.81	23.19 ± 2.95	<0.001	15.56 ± 2.01	15.62 ± 1.63	16.81 ± 2.41	15.89 ± 2.27	<0.001
SBP (mmHg)	127.11 ± 15.19	127.99 ± 13.07	129.50 ± 14.93	134.43 ± 15.99	<0.001	120.20 ± 17.22	126.83 ± 17.23	130.73 ± 17.42	134.63 ± 15.97	<0.001
DBP (mmHg)	75.43 ± 9.81	78.51 ± 7.65	79.02 ± 10.21	79.56 ± 10.79	<0.001	72.06 ± 10.29	73.30 ± 9.65	77.44 ± 9.96	76.29 ± 9.66	<0.001
Hypertension (*n*, %)	268 (19.2)	24 (22.6)	156 (21.4)	66 (35.1)	<0.001	123 (11.4%)	23 (26.1%)	83 (27.6%)	47 (40.9%)	<0.001
FBG (mmol/L)	5.35 (5.03 ~ 5.76)	5.74(5.40 ~ 6.10)	5.49 (5.14 ~ 5.94)	8.27 (7.43 ~ 9.47)	<0.001	5.12 (4.85 ~ 5.42)	5.50 (5.06 ~ 5.90)	5.39 (5.09 ~ 5.80)	8.10 (7.54 ~ 8.86)	<0.001
Diabetes (*n*, %)	108 (7.7)	0 (0)	0 (0)	188 (100.0)	<0.001	21 (1.9)	0 (0)	0 (0)	115 (100.0)	<0.001
TG (mmol/L)	1.27 (0.94 ~ 1.77)	2.02 (1.73 ~ 2.57)	1.87 (1.34 ~ 2.61)	2.18 (1.59 ~ 3.49)	<0.001	1.00 (0.78 ~ 1.36)	1.68 (1.13 ~ 2.15)	1.54 (1.27 ~ 2.16)	1.98 (0.97 ~ 3.03)	<0.001
TC (mmol/L)	4.77 (4.22 ~ 5.35)	4.92 (4.61 ~ 5.96)	4.92 (4.32 ~ 5.48)	4.83 (3.99 ~ 5.46)	<0.001	5.03 (4.52 ~ 5.68)	4.99 (4.64 ~ 5.69)	5.26 (4.57 ~ 5.88)	5.17 (4.11 ~ 5.97)	0.040
HDL-C(mmol/L)	1.22 (1.06 ~ 1.43)	1.10 (0.96 ~ 1.30)	1.07 (0.93 ~ 1.22)	1.02 (0.87 ~ 1.18)	<0.001	1.52 (1.32 ~ 1.77)	1.22 (1.16 ~ 1.41)	1.25 (1.13 ~ 1.41)	1.26 (1.07 ~ 1.42)	<0.001
LDL-C(mmol/L)	3.10 (2.54 ~ 3.64)	3.35 (3.14 ~ 4.11)	3.21 (2.65 ~ 3.74)	3.21 (2.65 ~ 3.74)	<0.001	3.22 (2.69 ~ 3.80)	3.14 (2.88 ~ 3.80)	3.43 (2.88 ~ 4.14)	3.32 (2.59 ~ 4.08)	0.005
Dyslipidemia (*n*, %)	598 (42.8)	101 (95.3)	456 (62.5)	134 (71.3)	<0.001	546 (50.6)	63 (71.6)	216 (71.8)	73 (63.5)	<0.001
ALT(IU/L)	17.00 (13.00 ~ 23.00)	17.00 (14.50 ~ 29.00)	26.00 (19.00 ~ 36.00)	26.00 (19.00 ~ 36.00)	<0.001	15.00 (11.00 ~ 19.00)	18.00 (13.00 ~ 24.00)	20.00 (16.00 ~ 28.00)	21.00 (17.00 ~ 34.00)	<0.001
AST(IU/L)	18.00 (15.00 ~ 21.00)	18.00 (15.00 ~ 23.00)	20.00(16.00 ~ 24.00)	20.00 (16.00 ~ 24.00)	<0.001	17.00 (14.00 ~ 19.70)	16.00 (15.00 ~ 20.00)	18.00 (15.00 ~ 21.00)	19.00(16.00 ~ 22.00)	<0.001
FIB-4 index	1.43 (1.08 ~ 1.83)	1.49 (1.26 ~ 1.56)	1.45 (1.16 ~ 2.27)	1.51 (1.03 ~ 2.00)	<0.001	1.06 (0.87 ~ 1.30)	1.05 (0.92 ~ 1.26)	1.13 (0.94 ~ 1.63)	1.19 (0.84 ~ 1.57)	0.004
NFS	−2.18 (−2.63 ~ −1.36)	−1.95 (−2.56 ~ −1.51)	−1.54 (−2.08 ~ −0.91)	−1.25(−1.73 ~ −0.58)	<0.001	−2.15 (−2.68 ~ −1.48)	−1.73 (−2.50 ~ −1.56)	−1.74 (−2.37 ~ −1.13)	−0.92 (−1.82 ~ −0.20)	<0.001
WBC (×10^9^/L)	5.96 (5.07 ~ 7.00)	6.46 (5.49 ~ 7.44)	6.44 (5.59 ~ 7.48)	6.44 (5.59 ~ 7.48)	<0.001	5.21 (4.47 ~ 6.24)	5.37 (4.72 ~ 6.03)	5.68 (4.91 ~ 6.60)	6.01 (5.55 ~ 6.76)	<0.001
NE (×10^9^/L)	3.29 (2.63 ~ 3.92)	3.35 (2.97 ~ 4.17)	3.48 (2.93 ~ 4.26)	3.48 (2.93 ~ 4.26)	<0.001	2.81 (2.25 ~ 3.48)	2.93 (2.57 ~ 3.74)	3.08 (2.48 ~ 3.80)	3.08 (2.89 ~ 3.69)	<0.001
LY(×10^9^/L)	2.04 (1.68 ~ 2.48)	2.38 (1.98 ~ 2.56)	2.32 (1.91 ~ 2.77)	2.32 (1.91 ~ 2.77)	<0.001	1.94 (1.63 ~ 2.28)	2.07 (1.91 ~ 2.28)	2.10 (1.75 ~ 2.41)	2.31 (1.83 ~ 3.00)	<0.001

**Table 2:** Baseline characteristics of the study population by SVR quartiles according to sex (*n* = 4,003).

Characteristics	Male (*n* = 2,420)				*P* for trend	Female (*n* = 1,583)				*P* for trend
	Q1 (*n* = 612)	Q2 (*n* = 596)	Q3 (*n* = 606)	Q4 (*n* = 606)		Q1 (*n* = 416)	Q2 (*n* = 378)	Q3 (*n* = 421)	Q4 (*n* = 368)	
Age (years)	61.52 ± 10.47	58.08 ± 8.04	56.42 ± 7.96	55.06 ± 7.48	<0.001	60.73 ± 9.25	56.92 ± 7.68	54.64 ± 7.83	51.72 ± 5.68	<0.001
Smoking (*n*, %)	337 (55.1)	359 (60.2)	378 (62.4)	307 (50.7)	0.224	11 (2.6)	10 (2.6)	2 (0.5)	10 (2.7)	0.482
Drinking (*n*, %)	192 (31.4)	226 (37.9)	240 (39.6)	189 (31.2)	0.882	31 (7.5)	12 (3.2)	9 (2.1)	6 (1.6)	<0.001
WC (cm)	97.47 ± 8.17	92.26 ± 5.81	88.32 ± 5.52	82.37 ± 4.96	<0.001	89.64 ± 7.83	84.69 ± 5.76	81.51 ± 5.24	76.38 ± 4.75	<0.001
BMI (kg/m^2^)	27.45 ± 2.82	25.69 ± 2.28	24.64 ± 2.16	22.95 ± 2.07	<0.001	26.12 ± 2.91	24.17 ± 2.07	22.76 ± 1.85	21.26 ± 1.83	<0.001
ASM (kg)	22.20 ± 3.26	22.76 ± 2.74	22.90 ± 2.98	22.59 ± 2.68	0.014	15.34 ± 2.24	15.90 ± 2.08	16.00 ± 2.15	16.10 ± 2.02	<0.001
VFA (cm^2^)	123.05 ± 25.21	92.81 ± 11.81	79.75 ± 10.88	59.41 ± 11.90	<0.001	140.32 ± 26.40	107.93 ± 15.32	85.33 ± 13.22	62.11 ± 11.27	<0.001
SBP (mmHg)	130.48 ± 14.89	129.90 ± 16.46	128.10 ± 14.62	125.28 ± 14.33	<0.001	130.39 ± 18.73	123.69 ± 17.38	123.27 ± 16.01	116.30 ± 16.71	<0.001
DBP (mmHg)	77.97 ± 10.28	78.26 ± 10.06	77.08 ± 9.57	74.58 ± 10.04	<0.001	76.41 ± 10.13	73.56 ± 10.60	73.42 ± 9.94	70.07 ± 9.93	<0.001
Hypertension (*n*, %)	139 (22.7)	147 (24.7)	125 (20.6)	103 (17.0)	0.004	117 (28.1)	68 (18.0)	58 (13.8)	33 (9.0)	<0.001
FPG (mmol/L)	5.62 (5.22 ~ 6.46)	5.56(5.14 ~ 6.15)	5.47 (5.06 ~ 6.02)	5.29 (5.02 ~ 5.69)	<0.001	5.45 (5.03 ~ 6.00)	5.27 (4.98 ~ 5.69)	5.24 (4.93 ~ 5.64)	5.04 (4.81 ~ 5.33)	<0.001
Diabetes (*n*, %)	96 (15.7)	81 (13.6)	61 (10.1)	58 (9.6)	<0.001	66 (15.9)	35 (9.3)	21 (5.0)	14 (3.8)	<0.001
MAFLD (*n*, %)	391 (65.6)	358 (58.5)	170 (28.1)	105 (17.3)	<0.001	206 (49.5)	160 (42.3)	94 (22.3)	44 (12.0)	<0.001
TG (mmol/L)	1.66 (1.18 ~ 2.22)	1.63 (1.18 ~ 2.30)	1.59 (1.09 ~ 2.40)	1.32 (0.91 ~ 1.94)	<0.001	1.28 (0.98 ~ 1.69)	1.25 (0.94 ~ 1.81)	1.17 (0.85 ~ 1.64)	0.94 (0.67 ~ 1.35)	<0.001
TC (mmol/L)	4.86 (4.14 ~ 5.32)	4.90 (4.30 ~ 5.51)	4.80 (4.26 ~ 5.42)	4.80 (4.25 ~ 5.38)	0.074	5.16 (4.62 ~ 5.77)	5.14 (4.54 ~ 5.71)	5.04 (4.47 ~ 5.75)	5.01 (4.49 ~ 5.62)	0.027
HDL-C(mmol/L)	1.10 (0.96 ~ 1.25)	1.10 (0.97 ~ 1.27)	1.16 (0.99 ~ 1.34)	1.27 (1.05 ~ 1.52)	<0.001	1.39 (1.22 ~ 1.62)	1.38 (1.23 ~ 1.59)	1.40 (1.18 ~ 1.67)	1.56 (1.31 ~ 1.87)	<0.001
LDL-C(mmol/L)	3.14 (2.53 ~ 3.68)	3.26 (2.65 ~ 3.76)	3.12 (2.59 ~ 3.75)	3.13 (2.46 ~ 3.60)	0.210	3.33 (2.79 ~ 4.00)	3.33 (2.72 ~ 3.84)	3.25 (2.82 ~ 3.80)	3.09 (2.58 ~ 3.71)	0.001
Dyslipidemia (*n*, %)	326 (53.3)	344 (57.7)	325 (53.6)	294 (48.5)	0.044	252 (60.6)	224 (59.3)	250 (59.4)	172 (46.7)	<0.001
ALT(IU/L)	21.00 (15.00 ~ 32.00)	21.00 (15.00 ~ 29.00)	20.00 (15.00 ~ 28.00)	17.00 (13.00 ~ 23.00)	<0.001	18.00 (13.25 ~ 24.00)	16.00 (13.00 ~ 21.00)	15.00 (12.00 ~ 20.00)	15.00 (11.00 ~ 19.75)	<0.001
AST(IU/L)	19.00 (16.00 ~ 23.00)	18.00 (15.00 ~ 22.00)	18.00 (15.00 ~ 23.00)	18.00 (15.00 ~ 21.00)	0.015	18.00 (15.00 ~ 21.00)	17.00 (14.00 ~ 20.00)	17.00 (14.00 ~ 20.00)	17.00 (14.00 ~ 20.00)	0.002
FIB-4 index	1.54 (1.24 ~ 2.16)	1.41 (1.09 ~ 1.74)	1.42 (1.04 ~ 1.86)	1.42 (1.06 ~ 1.90)	<0.001	1.19 (0.95 ~ 1.54)	1.05 (0.91 ~ 1.35)	1.05 (0.86 ~ 1.31)	1.02 (0.82 ~ 1.22)	<0.001
NFS	−1.21 (−1.83 ~ −0.59)	−1.24 (−1.87 ~ −0.64)	−1.55 (−2.20 ~ −0.88)	−1.60 (−2.15 ~ −1.13)	<0.001	−1.41 (−2.08 ~ −0.66)	−1.82 (−2.53 ~ −1.26)	−2.09 (−2.63 ~ −1.51)	−2.42 (−2.91 ~ −1.82)	<0.001
WBC (×10^9^/L)	6.27 (5.38 ~ 7.28)	6.39 (5.52 ~ 7.44)	6.19 (5.35 ~ 7.24)	5.75 (5.04 ~ 6.99)	<0.001	5.57 (4.93 ~ 6.67)	5.56 (4.75 ~ 6.54)	5.37 (4.60 ~ 6.31)	4.99 (4.37 ~ 5.89)	<0.001
NE (×10^9^/L)	3.46 (2.85 ~ 4.22)	3.36 (2.93 ~ 4.13)	3.37 (2.75 ~ 4.12)	3.24 (2.60 ~ 3.88)	<0.001	2.95 (2.47 ~ 3.59)	3.01 (2.46 ~ 3.65)	2.85 (2.29 ~ 3.64)	2.69 (2.18 ~ 3.36)	<0.001
LY(×10^9^/L)	2.27 (1.87 ~ 2.70)	2.21 (1.77 ~ 2.63)	2.11 (1.80 ~ 2.56)	2.04 (1.68 ~ 2.49)	<0.001	2.11 (1.75 ~ 2.55)	2.01 (1.72 ~ 2.35)	1.99 (1.67 ~ 2.29)	1.85 (1.56 ~ 2.13)	0.037

### Dose–response relationship between SVR values and MAFLD subtypes risk in male and female subjects

**Figure 3:**
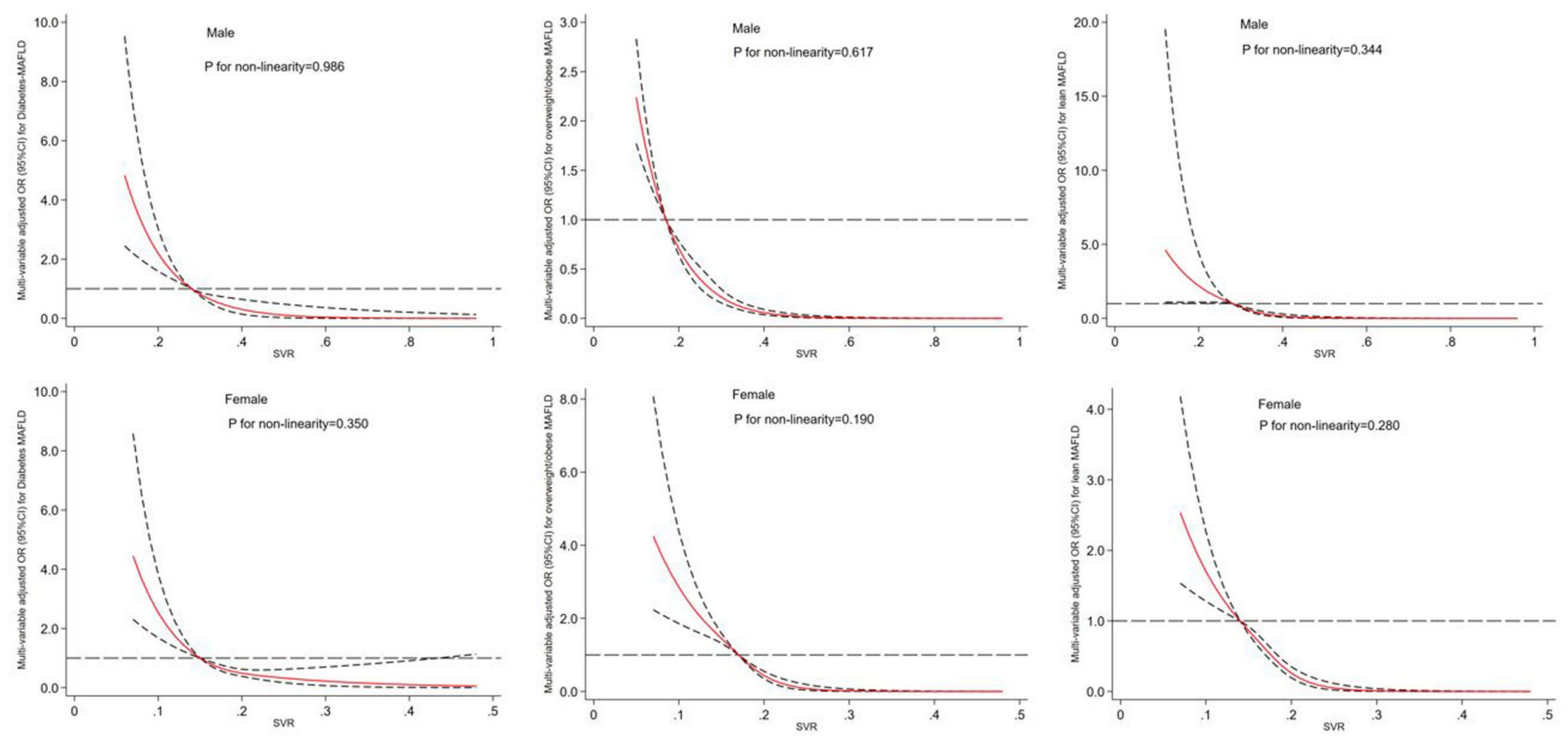
Dose–response relationship between SVR and risk of MAFLD subtypes in male and female. The restricted cubic spline regression analysis was adjusted for age, smoking, drinking, WC, BMI, FBG, hypertension, TG, TC, HDL-c, LDL-c, ALT, AST, WBC, NE and LY. The long dashed line represents OR is equal to 1, red line and the area between the short dashed lines means ORs and their 95%CI.

The results of a multivariable adjusted restricted cubic spline analysis showed that a decreasing SVR value significantly increased the risk of developing MAFLD subtypes in both males and females when SVR value was below its cut-off point. The cut-off points for males were 0.26 kg/cm^2^, 0.19 kg/cm^2^, and 0.24 kg/cm^2^, while for females they were 0.14 kg/cm^2^, 0.18 kg/cm^2^ and 0.15 kg/cm^2^. Above these cut-off points, a decreasing SVR value had a protective effect. Figure 3 illustrates the gender-specific dose–response relationships between SVR values and MAFLD subtypes risk, with the horizontal line representing the 5th, 35th, 65th, and 95th percentiles of SVR, and the red line representing the multivariable adjusted ORs for MAFLD with four knots located at the 5th, 35th, 65th, and 95th percentiles of SVR. We did not find a non-linear relationship between SVR and MAFLD subtypes (all *P*_for nonlinearity_ > 0.05).

### Association between SVR quartiles and risk of developing MAFLD subtypes and its fibrosis by gender

Table 3 demonstrates that, after adjusting for potential confounding factors such as age, sex, smoking, drinking, waist circumference, BMI, hypertension, dyslipidemia, ALT, AST, WBC, NE and LY, the first quartile of SVR significantly increased the prevalence of diabetes-MAFLD [2.15(1.25 ~ 3.71)], overweight-obese MAFLD [1.73(1.19 ~ 2.49)], and lean-MAFLD [2.38(1.49 ~ 3.80)] among all participants compared to the highest quartile of SVR (*P*_for trend_<0.001). Besides, we further explored the sex-specific association of SVR and MAFLD subtypes, the results indicated that middle-aged and elderly males with the lowest quartile of SVR had the highest risk of developing diabetes-MAFLD [2.96(1.48 ~ 5.93)], overweight/obese-MAFLD [1.91(1.26 ~ 2.88)], and lean-MAFLD [4.01(1.46 ~ 10.98)] compared to the fourth quartile as the reference group (*P* for trend<0.001). Additionally, females with the first quartile of SVR had a significantly higher risk of diabetes-MAFLD, overweight/obese-MAFLD, and lean-MAFLD with ORs [95% confidence interval (CI)] of 3.30(1.46 ~ 7.46), 4.48(1.91 ~ 10.49) and 2.53(1.19 ~ 5.37) respectively (*P*_for trend_<0.001). Additionally, the interaction effect of gender on the relationship between SVR and MAFLD subtypes was statistically significant (all *P*_for interaction_<0.001). Moreover, Tables 4, 5 showed that the lowest quartile of SVR is linked to an increased prevalence of MAFLD fibrosis in both males and females compared to the highest quartile when liver fibrosis is defined by either FIB-4 index or NFS (*P*_for trend_ < 0.001). Specifically, the first quartile of SVR was associated with a higher risk of MAFLD fibrosis in males (OR = 2.66, 95% CI: 1.88 ~ 3.76) and females (OR = 3.31, 95% CI: 2.08 ~ 5.26) when defined by FIB-4 index, and in males (OR = 1.97, 95% CI: 1.32 ~ 2.94) and females (OR = 3.84, 95% CI: 1.36 ~ 10.85) when defined by NFS. Besides, the association between SVR and MAFLD fibrosis was significantly different by gender when liver fibrosis was defined by NFS (*P*_for interaction_<0.001); however, no significant sex-specific difference was observed in the association between SVR and MAFLD fibrosis when liver fibrosis was defined by FIB-4 index (*P*_for interaction_>0.05).

**Table 3:** Adjusted associations between SVR quartiles and MAFLD subtypes by gender.

	Diabetes-MAFLD			Overweight/obese-MAFLD			Lean-MAFLD		
	Model 1	Model 2	Model 3	Model 1	Model 2	Model 3	Model 1	Model 2	Model 3
All participants									
Q1	2.17 (1.28 ~ 3.66)	2.19 (1.29 ~ 3.73)	**2.15 (1.25 ~ 3.71)**	1.75 (1.23 ~ 2.49)	1.74 (1.22 ~ 2.49)	**1.73 (1.19 ~ 2.49)**	2.66 (1.69 ~ 4.18)	2.30 (1.45 ~ 3.65)	**2.38 (1.49 ~ 3.80)**
Q2	2.02 (1.26 ~ 3.26)	1.97 (1.22 ~ 3.20)	1.69 (1.03 ~ 2.76)	2.13 (1.57 ~ 2.90)	2.09 (1.53 ~ 2.85)	1.86 (1.35 ~ 2.57)	2.15 (1.57 ~ 3.73)	2.46 (1.39 ~ 3.73)	2.27 (1.17 ~ 3.45)
Q3	1.09 (0.65 ~ 1.83)	1.04 (0.61 ~ 1.75)	0.92 (0.54 ~ 1.57)	1.46 (1.07 ~ 2.00)	1.44 (1.05 ~ 1.98)	1.31 (0.94 ~ 1.82)	2.16 (1.09 ~ 4.31)	1.91 (1.13 ~ 3.69)	1.95 (0.96 ~ 3.97)
Q4	1.00 (reference)	1.00 (reference)	1.00 (reference)	1.00 (reference)	1.00 (reference)	1.00 (reference)	1.00 (reference)	1.00 (reference)	1.00 (reference)
*P* for trend	<0.001	<0.001	<0.001	<0.001	<0.001	<0.001	<0.001	<0.001	<0.001
Male									
Q1	3.08 (1.57 ~ 6.02)	3.23 (1.64 ~ 6.35)	**2.96 (1.48 ~ 5.93)**	2.16 (1.46 ~ 3.20)	2.12 (1.43 ~ 3.15)	**1.91 (1.26 ~ 2.88)**	3.52 (1.37 ~ 9.01)	4.01 (1.48 ~ 10.88)	**4.01 (1.46 ~ 10.98)**
Q2	3.01 (1.61 ~ 5.63)	2.88 (1.54 ~ 5.41)	2.39 (1.26 ~ 4.54)	2.39 (1.69 ~ 3.38)	2.32 (1.63 ~ 3.29)	1.93 (1.34 ~ 2.78)	4.34 (2.18 ~ 8.65)	3.76 (1.80 ~ 7.85)	3.52 (1.66 ~ 7.46)
Q3	2.20 (1.18 ~ 4.09)	2.16 (1.15 ~ 4.06)	1.83 (0.96 ~ 3.48)	1.49 (1.06 ~ 2.10)	1.50 (1.06 ~ 2.12)	1.30 (0.91 ~ 1.86)	3.78 (2.15 ~ 6.66)	3.57 (1.96 ~ 6.51)	3.62 (1.96 ~ 6.68)
Q4	1.00 (reference)	1.00 (reference)	1.00 (reference)	1.00 (reference)	1.00 (reference)	1.00 (reference)	1.00 (reference)	1.00 (reference)	1.00 (reference)
*P* for trend	<0.001	<0.001	<0.001	<0.001	<0.001	<0.001	<0.001	<0.001	<0.001
Female									
Q1	5.77 (2.67 ~ 12.43)	5.85 (2.67 ~ 12.80)	**3.30 (1.46 ~ 7.46)**	5.34 (2.38 ~ 12.00)	6.11 (2.69 ~ 13.86)	**4.48 (1.91 ~ 10.49)**	2.45 (1.19 ~ 5.05)	2.76 (1.33 ~ 5.74)	**2.53 (1.19 ~ 5.37)**
Q2	2.94 (1.35 ~ 6.41)	3.11 (1.42 ~ 6.83)	1.97 (0.85 ~ 4.55)	6.56 (3.02 ~ 14.22)	7.16 (3.27 ~ 15.68)	5.62 (2.48 ~ 12.72)	2.04 (1.10 ~ 3.78)	2.15 (1.15 ~ 4.02)	2.04 (1.08 ~ 3.89)
Q3	1.28 (0.54 ~ 3.01)	1.32 (0.55 ~ 3.14)	0.90 (0.36 ~ 2.28)	3.78 (1.73 ~ 8.24)	3.83 (1.74 ~ 8.44)	3.01 (1.32 ~ 6.84)	1.62 (0.66 ~ 3.97)	1.96 (0.79 ~ 4.85)	1.62 (0.65 ~ 4.04)
Q4	1.00 (reference)	1.00 (reference)	1.00 (reference)	1.00 (reference)	1.00 (reference)	1.00 (reference)	1.00 (reference)	1.00 (reference)	1.00 (reference)
*P* for trend	<0.001	<0.001	<0.001	<0.001	<0.001	<0.001	<0.001	<0.001	<0.001
*P* for interaction	<0.001	<0.001	<0.001	<0.001	<0.001	<0.001	<0.001	<0.001	<0.001

Model 1: Age, smoking, drinking, WC and BMI. Model 2: Model 1 + FBG, hypertension and dyslipidemia. Model 3: Model 2 + ALT, AST, WBC, NE and LY.

**Table 4:** Logistic regression analysis to identify the association between SVR quartiles and MAFLD fibrosis (FIB-4 index) by gender.

	Total (*n*, %)	MAFLD with Fibrosis (*n*, %)	Model 1		Model 2		Model 3	
Liver fibrosis defined by FIB-4 index			OR (95%CI)	*P*-value	OR (95%CI)	*P*-value	OR (95%CI)	*P*-value
Male	*n* = 2,420	*n* = 546						
Q1	612 (25.3)	208 (38.1)	2.48 (1.76 ~ 3.48)	<0.001	2.49 (1.77 ~ 3.49)	<0.001	**2.66 (1.88 ~ 3.76)**	<0.001
Q2	596 (24.6)	201 (36.8)	1.95 (1.47 ~ 2.58)	<0.001	1.91 (1.44 ~ 2.53)	<0.001	2.13 (1.60 ~ 2.84)	<0.001
Q3	606 (25.1)	86 (15.8)	1.32 (1.03 ~ 1.70)	0.029	1.31 (1.02 ~ 1.69)	0.034	1.42 (1.10 ~ 1.84)	0.008
Q4	606 (25.0)	51 (9.3)	1.00 (reference)		1.00 (reference)		1.00 (reference)	
*P* for trend			<0.001		<0.001		<0.001	
Female	*n* = 1,583	*n* = 229						
Q1	416 (26.3)	116 (50.7)	2.32 (1.50 ~ 3.59)	<0.001	2.53 (1.63 ~ 3.93)	<0.001	**3.31 (2.08 ~ 5.26)**	<0.001
Q2	378 (23.9)	76 (33.2)	2.19 (1.50 ~ 3.20)	<0.001	2.33 (1.59 ~ 3.41)	<0.001	2.96 (1.98 ~ 4.42)	<0.001
Q3	421 (26.6)	31 (13.5)	1.48 (1.04 ~ 2.12)	0.030	1.54 (1.08 ~ 2.21)	0.018	1.80 (1.24 ~ 2.61)	0.002
Q4	368 (23.2)	6 (2.6)	1.00 (reference)		1.00 (reference)		1.00 (reference)	
*P* for trend			<0.001		<0.001		<0.001	
*P* for interaction			0.315		0.255		0.236	

Model 1: Age, smoking, drinking, WC and BMI. Model 2: Model 1 + FBG, hypertension and dyslipidemia. Model 3: Model 2 + ALT, AST, WBC, NE and LY.

**Table 5:** Logistic regression analysis to identify the association between SVR quartiles and MAFLD fibrosis (NFS) by gender.

	Total (*n*, %)	MAFLD with Fibrosis (*n*, %)	Model 1		Model 2		Model 3	
Liver fibrosis defined by NFS			OR (95%CI)	*P*-value	OR (95%CI)	*P*-value	OR (95%CI)	*P*-value
Male	*n* = 2,420	*n* = 528						
Q1	612 (25.3)	196 (37.1)	1.82 (1.27 ~ 2.63)	0.001	1.74 (1.20 ~ 2.52)	0.004	**1.97 (1.32 ~ 2.94)**	0.001
Q2	596 (24.6)	187 (35.4)	1.50 (1.05 ~ 2.16)	0.027	1.57 (1.09 ~ 2.27)	0.016	1.76 (1.18 ~ 2.62)	0.006
Q3	606 (25.1)	84 (15.9)	0.88 (0.61 ~ 1.27)	0.492	0.91 (0.63 ~ 1.32)	0.607	1.07 (0.72 ~ 1.59)	0.750
Q4	606 (25.0)	61 (11.6)	1.00 (reference)		1.00 (reference)		1.00 (reference)	
*P* for trend			<0.001		<0.001		<0.001	
Female	*n* = 1,583	*n* = 203						
Q1	416 (26.3)	123 (60.6)	4.08 (1.46 ~ 11.35)	0.007	4.58 (1.64 ~ 12.84)	0.004	**3.84 (1.36 ~ 10.85)**	0.011
Q2	378 (23.9)	59 (29.1)	3.78 (1.42 ~ 10.07)	0.008	4.00 (1.49 ~ 10.72)	0.006	3.28 (1.21 ~ 8.90)	0.020
Q3	421 (26.6)	16 (7.9)	1.65 (0.58 ~ 4.69)	0.348	1.64 (0.57 ~ 4.67)	0.358	1.38 (0.48 ~ 4.01)	0.550
Q4	368 (23.2)	5 (2.5)	1.00 (reference)		1.00 (reference)		1.00 (reference)	
*P* for trend			<0.001		<0.001		<0.001	
*P* for interaction			<0.001		<0.001		<0.001	

Model 1: Age, smoking, drinking, WC and BMI. Model 2: Model 1 + FBG, hypertension and dyslipidemia. Model 3: Model 2 + ALT, AST, WBC, NE and LY.

## Discussion

Our findings demonstrate that in this single-center retrospective study of 4,003 middle-aged participants, all participants with MAFLD subtypes had a significantly lower SVR than those without MAFLD, and increasing quartiles of FIB-4 and NFS were also linked with a lower SVR, regardless of gender. Moreover, multivariable regression analysis indicated that low SVR significantly increased the prevalence of MAFLD subtypes including diabetes-MAFLD, overweight/obese-MAFLD and lean-MAFLD in both males and females. Moreover, we observed a close relationship between decreasing SVR and the risk of MAFLD with the presence of fibrosis. To our best knowledge, this is the first research to date that has examined the sex-specific associations of SVR and MAFLD subtypes and its hepatic condition.

Previous research has suggested that obesity is a risk factor which can exacerbate chronic liver disease, and that reduced skeletal muscle mass can increase the risk of developing NAFLD, as well as having an impact on the post-surgical outcome of those who have had a liver transplant (11, 27–30). Furthermore, a decrease in muscle mass can lead to an increase in visceral fat, which plays a role in the onset and progression of MAFLD. Recent study has highlighted a relationship between low skeletal muscle mass and the risk of NAFLD, adjusting for BMI or body weight, but not taking visceral fat into consideration (27). SVR, which considers both muscle mass and visceral fat, has been used to identify sarcopenic visceral obesity, and has been associated with NAFLD for both sexes (14). In contrast to a cross-sectional study of Chinese patients with type 2 diabetes which revealed a connection between SVR and ultrasonography-defined NAFLD, but only for women (31), our research concentrated on the association between SVR and MAFLD subtypes in middle-aged and older individuals, who possess a greater risk of developing chronic liver disease than younger individuals. In line with our study, another recent study also demonstrated that decreased muscle mass coupled with excessive visceral adipose is closely related to an increased risk of exacerbating NAFLD pathophysiology referring to moderate-to-severe steatosis and that of advanced fibrosis (14). In our research, the effects of SVR on the risk of diabetes-MAFLD and overweight-MAFLD were more prominent in women than in men, even though the absolute occurrence of MAFLD was much lower in women than in men. Research on the prevalence of fibrotic non-alcoholic steatohepatitis (NASH) in the United States population recently revealed that probable fibrotic NASH was present in 8.4% of the population (95% CI 8.0–8.8), with more cases found in males and Hispanic individuals (32). Women, particularly premenopausal women, tend to have fat distributed more beneficially, such as in the gluteofemoral region and subcutaneous area, as opposed to men who store fat mainly in the visceral area. Middle-aged and elderly females in our study were almost in postmenopausal state, and were thereby at a higher risk of MAFLD. Previous study also demonstrated that, independently from BMI, an android fat deposition pattern is associated with increased prevalence of NAFLD in both sexes (33). Our study also showed that women had less appendicular skeletal muscle mass than men (15.64 ± 1.85 kg vs. 21.86 ± 2.89 kg, *P* <0.001), indicating that BMI may not be a reliable measure of metabolic risk in women. We suggest that sarcopenic visceral obesity may be a better indicator of metabolic risk in women. To gain a better understanding of the different effects of SVR on MAFLD risk between genders, further research is necessary to accurately assess fat distribution and skeletal muscle mass. A cohort study revealed that the relationship between SVR and NAFLD was more prominent in non-obese people than in obese individuals, and suggested that low SVR is a supplementary index to traditional measures of obesity when assessing the risk of NAFLD (34).

It is known that when skeletal muscle mass is reduced and visceral adipose tissue accumulates, the insulin-mediated ability of skeletal muscle and adipose tissue to use or store blood glucose is impaired. This decrease in whole-body insulin-mediated glucose uptake and the associated insulin resistance can cause persistent muscle loss, and may even be a factor in the development of MAFLD (35). Our research showed that middle-aged and older males with low SVR were more likely to develop MAFLD than females. We also found that males in the lowest quartile of SVR had the highest odds of developing lean-MAFLD compared to other MAFLD subtypes. Additionally, the predominant obesity phenotype in the Asian population is abdominal obesity, which is characterized by an excess of visceral adipose tissue deposition. Our study revealed that middle-aged and older females also had a higher level of visceral fat area than males (99.78 ± 27.62 cm^2^ vs. 86.06 ± 25.12 cm^2^, *P*<0.001), and were more likely to develop diabetes-MAFLD or overweight/obese MAFLD. A study has demonstrated that the prevalence of NAFLD is significantly higher in postmenopausal females than premenopausal females (36). This suggests that estrogen may act as a protective biomarker against the development of hepatic steatosis (37, 38). This hypothesis is supported by a cohort study in patients with NAFLD, which reported a higher risk of severe fibrosis in males than premenopausal females, but similar risk levels between males and postmenopausal females, which is in line with our findings. This indicates that estrogen may have a protective effect against liver fibrosis in NAFLD (39).

Our research has several limitations which should be considered. This single-center retrospective study design does not provide sufficient evidence to determine the causal relationship between SVR and MAFLD subtypes and its hepatic condition. To confirm our findings, more studies with larger samples and a wider range of participants in multi-centers are required in a longitudinal manner. Second, Sarcopenia is not only characterized by a decrease in muscle mass, but also involves muscle function including reduced hand-grip strength and slower gait speed. However, the diagnosis of sarcopenic obesity necessitates combining SVR and muscle function, but the latter was not measured in our study. Besides, we did not include any specific data regarding physical activities, menopausal status, sex hormone levels, or the utilization of estrogen and progestogen medications.

Finally, A study recently investigated the epidemiological effect of the definition of steatotic liver disease (SLD) proposed by a multi-society Delphi consensus statement, and found that there was a high degree of agreement between metabolic dysfunction-associated steatotic liver disease (MASLD) and the definition of MAFLD previously proposed. However, due to the lack of data including controlled attenuation parameter and liver stiffness measurement, the study could not determine if SVR is still significantly associated with MASLD risk by gender (40).

## Conclusion

Our study concluded that a decrease in SVR (appendicular skeletal muscle mass divided by visceral fat area) is significantly associated with an increased prevalence of MAFLD subtypes and liver fibrosis in middle-aged and older persons of both genders. Notably, there was a moderating effect of gender on the relationship between SVR and diabetes-MAFLD as well as overweight/obese MAFLD. Compared to females, males with low SVR had a greater chance of having lean-MAFLD. Furthermore, because of the lower SVR in females, middle-aged and elderly females were more likely to have diabetes-MAFLD and overweight-obese MAFLD than males. Additionally, females with the lowest quartile of SVR also had a higher probability of having MAFLD with fibrosis than males. To address this issue, healthcare professionals should promote hepatic rehabilitation through diet and exercise therapies to reduce abnormal body composition. To gain a better understanding of the relationship between SVR and the risk and progression of MAFLD subtypes, future research should include larger sample sizes and different age and ethnic groups, both from a prospective and mechanistic perspective.

## Data availability statement

The raw data supporting the conclusions of this article will be made available by the authors, without undue reservation.

## Ethics statement

The studies involving humans were approved by the Ethics and Research Committee of Health Examination Center of Wuxi People’s Hospital Affiliated to Nanjing Medical University. The studies were conducted in accordance with the local legislation and institutional requirements. The ethics committee/institutional review board waived the requirement of written informed consent for participation from the participants or the participants’ legal guardians/next of kin because personal data was anonymized in order to protect the privacy of patients; statistical analysis was conducted in a confidential manner and was only utilized for scientific objectives.

## Author contributions

XY conceived and designed the study and supervised and critically reviewed the original manuscript and contributed to statistical analyzes. MX and YN collected the clinical and demographic data. XZ and YZ were responsible for data validation and reviewed and designed visualizations (tables and figures). MX drafted the original manuscript with YN. YZ contributed to project administration and funding acquisition. All authors have read and agreed to the published version of the manuscript.

## References

[1] Younossi Z Anstee QM Marietti M Hardy T Henry L Eslam M. Global burden of NAFLD and NASH: trends, predictions, risk factors and prevention. Nat Rev Gastroenterol Hepatol. (2018) 15:11–20. doi: 10.1038/nrgastro.2017.109, PMID: 28930295

[2] Eslam M Sarin SK Wong VW Fan JG Kawaguchi T Ahn SH. The Asian Pacific Association for the Study of the liver clinical practice guidelines for the diagnosis and management of metabolic associated fatty liver disease. Hepatol Int. (2020) 14:889–919. doi: 10.1007/s12072-020-10094-2, PMID: 33006093

[3] Eslam M Sanyal AJ George J. MAFLD: a consensus-driven proposed nomenclature for metabolic associated fatty liver disease. Gastroenterology. (2020) 158:1999–2014. doi: 10.1053/j.gastro.2019.11.312, PMID: 32044314

[4] Pipitone RM Ciccioli C Infantino G La Mantia C Parisi S Tulone A. MAFLD: a multisystem disease. Ther Adv Endocrinol Metab. (2023) 14:1859452467. doi: 10.1177/20420188221145549, PMID: 36726391 PMC9885036

[5] Byrne CD Targher G. NAFLD as a driver of chronic kidney disease. J Hepatol. (2020) 72:785–801. doi: 10.1016/j.jhep.2020.01.013, PMID: 32059982

[6] Mantovani A Petracca G Beatrice G Csermely A Tilg H Byrne CD. Non-alcoholic fatty liver disease and increased risk of incident extrahepatic cancers: a meta-analysis of observational cohort studies. Gut. (2022) 71:778–88. doi: 10.1136/gutjnl-2021-324191, PMID: 33685968

[7] Mantovani A Csermely A Petracca G Beatrice G Corey KE Simon TG. Non-alcoholic fatty liver disease and risk of fatal and non-fatal cardiovascular events: an updated systematic review and meta-analysis. Lancet Gastroenterol Hepatol. (2021) 6:903–13. doi: 10.1016/S2468-1253(21)00308-3, PMID: 34555346

[8] Jakobsen MU Berentzen T Sorensen TI Overvad K. Abdominal obesity and fatty liver. Epidemiol Rev. (2007) 29:77–87. doi: 10.1093/epirev/mxm002, PMID: 17478441

[9] Fabbrini E Sullivan S Klein S. Obesity and nonalcoholic fatty liver disease: biochemical, metabolic, and clinical implications. Hepatology. (2010) 51:679–89. doi: 10.1002/hep.23280, PMID: 20041406 PMC3575093

[10] Shuster A Patlas M Pinthus JH Mourtzakis M. The clinical importance of visceral adiposity: a critical review of methods for visceral adipose tissue analysis. Br J Radiol. (2012) 85:1–10. doi: 10.1259/bjr/38447238, PMID: 21937614 PMC3473928

[11] Cai C Song X Chen Y Chen X Yu C. Relationship between relative skeletal muscle mass and nonalcoholic fatty liver disease: a systematic review and meta-analysis. Hepatol Int. (2020) 14:115–26. doi: 10.1007/s12072-019-09964-1, PMID: 31290072 PMC6994447

[12] Klip A Paquet MR. Glucose transport and glucose transporters in muscle and their metabolic regulation. Diabetes Care. (1990) 13:228–43. doi: 10.2337/diacare.13.3.228, PMID: 2407478

[13] Severinsen M Pedersen BK. Muscle-organ crosstalk: the emerging roles of myokines. Endocr Rev. (2020) 41:594–609. doi: 10.1210/endrev/bnaa016, PMID: 32393961 PMC7288608

[14] Shida T Akiyama K Oh S Sawai A Isobe T Okamoto Y. Skeletal muscle mass to visceral fat area ratio is an important determinant affecting hepatic conditions of non-alcoholic fatty liver disease. J Gastroenterol. (2018) 53:535–47. doi: 10.1007/s00535-017-1377-3, PMID: 28791501

[15] Janssen I Heymsfield SB Baumgartner RN Ross R. Estimation of skeletal muscle mass by bioelectrical impedance analysis. J Appl Physiol (1985). (2000) 89:465–71. doi: 10.1152/jappl.2000.89.2.465, PMID: 10926627

[16] Weng J Ji L Jia W Lu J Zhou Z Zou D. Standards of care for type 2 diabetes in China. Diabetes Metab Res Rev. (2016) 32:442–58. doi: 10.1002/dmrr.2827, PMID: 27464265 PMC5108436

[17] Chen Y Hu S Wu L Fang X Xu W Shen G. Clinical practice guidelines for hypertension in China: a systematic review of the methodological quality. BMJ Open. (2015) 5:e8099. doi: 10.1136/bmjopen-2015-008099, PMID: 26179649 PMC4513449

[18] Kang SH Cho Y Jeong SW Kim SU Lee JW. From nonalcoholic fatty liver disease to metabolic-associated fatty liver disease: big wave or ripple? Clin Mol Hepatol. (2021) 27:257–69. doi: 10.3350/cmh.2021.0067, PMID: 33751877 PMC8046627

[19] Farrell GC Chitturi S Lau GK Sollano JD. Guidelines for the assessment and management of non-alcoholic fatty liver disease in the Asia-Pacific region: executive summary. J Gastroenterol Hepatol. (2007) 22:775–7. doi: 10.1111/j.1440-1746.2007.05002.x, PMID: 17565629

[20] Rajindrajith S Pathmeswaran A Jayasinghe C Kottahachchi D Kasturiratne A De Silva ST. Non-alcoholic fatty liver disease and its associations among adolescents in an urban, Sri Lankan community. BMC Gastroenterol. (2017) 17:135. doi: 10.1186/s12876-017-0677-7, PMID: 29187144 PMC5708084

[21] Chen YL Li H Li S Xu Z Tian S Wu J. Prevalence of and risk factors for metabolic associated fatty liver disease in an urban population in China: a cross-sectional comparative study. BMC Gastroenterol. (2021) 21:212. doi: 10.1186/s12876-021-01782-w, PMID: 33971822 PMC8111711

[22] Sohn W Kwon HJ Chang Y Ryu S Cho YK. Liver fibrosis in asians with metabolic dysfunction-associated fatty liver disease. Clin Gastroenterol Hepatol. (2022) 20:e1135–48. doi: 10.1016/j.cgh.2021.06.042, PMID: 34224877

[23] Lee H Lim TS Kim SU Kim HC. Long-term cardiovascular outcomes differ across metabolic dysfunction-associated fatty liver disease subtypes among middle-aged population. Hepatol Int. (2022) 16:1308–17. doi: 10.1007/s12072-022-10407-7, PMID: 36070124

[24] Park H Yoon EL Kim M Lee J Kim JH Cho S. Comparison of diagnostic performance between FIB-4 and NFS in metabolic-associated fatty liver disease era. Hepatol Res. (2022) 52:247–54. doi: 10.1111/hepr.13737, PMID: 34841632

[25] Vallet-Pichard A Mallet V Nalpas B Verkarre V Nalpas A Dhalluin-Venier V. FIB-4: an inexpensive and accurate marker of fibrosis in HCV infection. Comparison with liver biopsy and fibrotest. Hepatology. (2007) 46:32–6. doi: 10.1002/hep.21669, PMID: 17567829

[26] Angulo P Hui JM Marchesini G Bugianesi E George J Farrell GC. The NAFLD fibrosis score: a noninvasive system that identifies liver fibrosis in patients with NAFLD. Hepatology. (2007) 45:846–54. doi: 10.1002/hep.21496, PMID: 17393509

[27] Kim G Lee SE Lee YB Jun JE Ahn J Bae JC. Relationship between relative skeletal muscle mass and nonalcoholic fatty liver disease: a 7-year longitudinal study. Hepatology. (2018) 68:1755–68. doi: 10.1002/hep.30049, PMID: 29679374

[28] Koo BK Kim D Joo SK Kim JH Chang MS Kim BG. Sarcopenia is an independent risk factor for non-alcoholic steatohepatitis and significant fibrosis. J Hepatol. (2017) 66:123–31. doi: 10.1016/j.jhep.2016.08.019, PMID: 27599824

[29] Hassan EA Makhlouf NA Ibrahim ME Dabbous HM Salah MA Aboalam HS. Impact of sarcopenia on short-term complications and survival after liver transplant. Exp Clin Transplant. (2022) 20:917–24. doi: 10.6002/ect.2022.0293, PMID: 36409051

[30] Kumar VV Kothakota SR Nair AK Sasidharan M Kareem H Kanala J. Impact of sarcopenia on post-liver transplant morbidity and mortality in cirrhotic patients. Indian J Gastroenterol. (2022) 41:440–5. doi: 10.1007/s12664-022-01262-3, PMID: 36342633

[31] Su X Xu J Zheng C. The relationship between non-alcoholic fatty liver and skeletal muscle mass to visceral fat area ratio in women with type 2 diabetes. BMC Endocr Disord. (2019) 19:76. doi: 10.1186/s12902-019-0404-1, PMID: 31315613 PMC6637487

[32] Ciardullo S Perseghin G. Trends in prevalence of probable fibrotic non-alcoholic steatohepatitis in the United States, 1999-2016. Liver Int. (2023) 43:340–4. doi: 10.1111/liv.15503, PMID: 36565051

[33] Ciardullo S Oltolini A Cannistraci R Muraca E Perseghin G. Sex-related association of nonalcoholic fatty liver disease and liver fibrosis with body fat distribution in the general US population. Am J Clin Nutr. (2022) 115:1528–34. doi: 10.1093/ajcn/nqac059, PMID: 35244676

[34] Cho Y Chang Y Ryu S Jung HS Kim CW Oh H. Skeletal muscle mass to visceral fat area ratio as a predictor of NAFLD in lean and overweight men and women with effect modification by sex. Hepatol Commun. (2022) 6:2238–52. doi: 10.1002/hep4.1975, PMID: 35503803 PMC9426405

[35] Kuhl J Hilding A Ostenson CG Grill V Efendic S Bavenholm P. Characterisation of subjects with early abnormalities of glucose tolerance in the Stockholm diabetes prevention programme: the impact of sex and type 2 diabetes heredity. Diabetologia. (2005) 48:35–40. doi: 10.1007/s00125-004-1614-1, PMID: 15619073

[36] Clark JM Brancati FL Diehl AM. Nonalcoholic fatty liver disease. Gastroenterology. (2002) 122:1649–57. doi: 10.1053/gast.2002.33573, PMID: 12016429

[37] Yang JD Abdelmalek MF Pang H Guy CD Smith AD Diehl AM. Gender and menopause impact severity of fibrosis among patients with nonalcoholic steatohepatitis. Hepatology. (2014) 59:1406–14. doi: 10.1002/hep.26761, PMID: 24123276 PMC3966932

[38] Venetsanaki V Polyzos SA. Menopause and non-alcoholic fatty liver disease: a review focusing on therapeutic perspectives. Curr Vasc Pharmacol. (2019) 17:546–55. doi: 10.2174/1570161116666180711121949, PMID: 29992886

[39] Rettberg JR Yao J Brinton RD. Estrogen: a master regulator of bioenergetic systems in the brain and body. Front Neuroendocrinol. (2014) 35:8–30. doi: 10.1016/j.yfrne.2013.08.001, PMID: 23994581 PMC4024050

[40] Ciardullo S Carbone M Invernizzi P Perseghin G. Exploring the landscape of steatotic liver disease in the general US population. Liver Int. (2023) 43:2425–33. doi: 10.1111/liv.15695, PMID: 37592856

